# Differential promoter methylation of kinesin family member 1a in plasma is associated with breast cancer and DNA repair capacity

**DOI:** 10.3892/or.2014.3262

**Published:** 2014-06-13

**Authors:** RAFAEL GUERRERO-PRESTON, TAL HADAR, KIMBERLY LASKIE OSTROW, ETHAN SOUDRY, MIGUEL ECHENIQUE, CARMEN ILI-GANGAS, GABRIELA PÉREZ, JIMENA PEREZ, PRISCILLA BREBI-MIEVILLE, JOSÉ DESCHAMPS, LUISA MORALES, MANUEL BAYONA, DAVID SIDRANSKY, JAIME MATTA

**Affiliations:** 1 Department of Otolaryngology - Head and Neck Cancer Research Division, Johns Hopkins School of Medicine, Baltimore, MD 21231, USA; 2 Department of Obstetrics and Gynecology, University of Puerto Rico School of Medicine, San Juan 00927, Puerto Rico; 3 Cancer Center, Auxilio Mutuo Hospital, San Juan, Puerto Rico; 4 Molecular Pathology Laboratory, Department of Pathology, CEGIN-BIOREN, School of Medicine, Universidad de La Frontera, Temuco, Chile; 5 Department of Pharmacology and Toxicology, Ponce School of Medicine and Health Sciences, Ponce 00732-7004, Puerto Rico; 6 Public Health Program, Ponce School of Medicine and Health Sciences, Ponce 00732-7004, Puerto Rico

**Keywords:** epigenetics, epigenetic biomarker panel, breast cancer, *KIF1A*, *OGDHL*, *FKBP4*, *VGF*, *MAL* promoter methylation, DNA repair capacity

## Abstract

Methylation alterations of CpG islands, CpG island shores and first exons are key events in the formation and progression of human cancer, and an increasing number of differentially methylated regions and genes have been identified in breast cancer. Recent studies of the breast cancer methylome using deep sequencing and microarray platforms are providing a novel insight on the different roles aberrant methylation plays in molecular subtypes of breast cancer. Accumulating evidence from a subset of studies suggests that promoter methylation of tumor-suppressor genes associated with breast cancer can be quantified in circulating DNA. However, there is a paucity of studies that examine the combined presence of genetic and epigenetic alterations associated with breast cancer using blood-based assays. Dysregulation of DNA repair capacity (DRC) is a genetic risk factor for breast cancer that has been measured in lymphocytes. We isolated plasma DNA from 340 participants in a breast cancer case control project to study promoter methylation levels of five genes previously shown to be associated with breast cancer in frozen tissue and in cell line DNA: *MAL*, *KIF1A*, *FKBP4*, *VGF* and *OGDHL*. Methylation of at least one gene was found in 49% of the cases compared to 20% of the controls. Three of the four genes had receiver characteristic operator curve values of ≥0.50: *MAL* (0.64), *KIF1A* (0.51) and *OGDHL* (0.53). *KIF1A* promoter methylation was associated with breast cancer and inversely associated with DRC. This is the first evidence of a significant association between genetic and epigenetic alterations in breast cancer using blood-based tests. The potential diagnostic utility of these biomarkers and their relevance for breast cancer risk prediction should be examined in larger cohorts.

## Introduction

According to the World Health Organization, more than 1.2 million women worldwide will be diagnosed with breast cancer (BC) this year. Worldwide, the incidence of BC is increasing by 3.1% annually (1980 to 2010 statistics) (1). BC is the most common malignancy in women, accounting for 23% of all female malignancies (2,3). BC is also the leading cause of cancer-related death among Puerto Rican women. Data from the Puerto Rico Cancer Registry show that BC accounted for 30.3% of all female cancers between 2005 and 2009 and 18.8% of all female cancer-related deaths between 2004 and 2008 (4). Mammography’s limitations in early detection of BC have been documented, particularly in women with pre-menopausal BC (5).

BC is a complex disease resulting from a combination of genetic, epigenetic and environmental factors (6). Aberrant promoter methylation of several known or putative tumor-suppressor genes (TSGs) occurs frequently during the pathogenesis of human cancer, including BC. Methylated TSGs are promising biomarkers for cancer screening that can be measured in plasma (7,8). Similarly, dysregulation of DNA repair pathways predisposes cells to accumulating damage and eventually mutations. Epidemiological studies using functional repair assays in lymphocytes or other cell types have demonstrated that DNA repair capacity (DRC) varies greatly among individuals and that a low repair capacity is a significant risk factor for the development of several types of cancers, including BC (9,10). Our previous studies have shown that a low DRC is an important risk factor for BC in Puerto Rican women (11,12). Few studies have examined the combined contribution of genetic and epigenetic alterations associated with BC using blood-based assays. To test the association of *MAL*, *KIF1A*, *FKBP4*, *VGF*, and *OGDHL* promoter methylation with BC, we obtained plasma DNA from 340 participants in a case-control BC study (9). Since women with BC in this cohort have an average decrease of 60% in their DCR levels we also performed a subset analysis to examine the association between promoter methylation of *MAL*, *KIF1A*, *FKBP4* and *OGDHL* and DRC levels.

## Materials and methods

### Study design

Plasma DNA from 340 participants, randomly selected from an incident-case case-control study of 1,186 Puerto Rican resident women representing ~83% of the island’s municipalities (counties) was obtained after IRB approval from the Institutional Review Boards (IRB) of the Johns Hopkins School of Medicine and the Ponce School of Medicine and Health Sciences. The participants were selected with the random selection subroutine of SPSS: Select Cases, Random Sample of Cases (IBM SPSS, version 22; Chicago IL, USA).

### Study population

All participants were female residents of Puerto Rico, recruited primarily from private practice offices of oncologists, gynecologists and surgeons in the cities of San Juan, Ponce, Salinas and Yauco. The age of the participants ranged from 30 to 89 years. A total of 502 BC cases and 684 controls, prospectively recruited over 7 years (2006 to 2013), were included in the original study sample (11).

Cases were women with a diagnosis of primary BC recruited consecutively from individuals visiting gynecological and primary care medical offices in Puerto Rico. The inclusion criteria for cases were as follows: patients who were recently diagnosed with primary BC, confirmed by histopathology and not receiving chemotherapy, blood transfusions, or radiotherapy. Patients with BC secondary to other types of cancer were not included. The pathology report from each case was reviewed to confirm the diagnosis.

Controls were women without BC recruited consecutively from individuals visiting gynecological and primary care medical offices in Puerto Rico for their routine mammograms and other types of wellness screenings. The two inclusion criteria for controls were: have a normal clinical breast examination performed by their primary physicians and normal mammogram result, both no more than six months prior to enrollment. Controls were recruited from the same population (clinics, physician offices and hospitals) where the cases came from; if they were to eventually develop BC, they would be treated in the same clinics where the cases were recruited. These selection criteria minimized selection bias. Women with BC secondary to another type of cancer were excluded from the present study.

BC patients and controls completed informed consent and HIPAA forms, as well as a self-administered questionnaire. The seven-page epidemiological questionnaire was used to gather epidemiological information on risk factors in relation to family history, genetic, hormonal and lifestyle factors. The characteristics of study participants including age, body mass index (BMI), family history of BC, age of menopause, alcohol consumption, smoking habits, consumption of multivitamins and DRC are summarized in Table I.

### DNA methylation analysis

DNA from 340 participants was extracted from 500 μl of plasma. Genomic DNA from 280 (81%) patients had 260/280 ratios >1.7 and amplified β-actin, thus meeting our stringent QC filter. We randomly divided the participants into a discovery set (20 cases and 20 controls) and a validation set (154 cases and 86 controls). Briefly, bisulfite-modified DNA was used as template for fluorescence-based real-time PCR, as previously described (13). Fluorogenic PCR reactions were carried out in a reaction volume of 20 ml consisting of 600 nmol/l of each primer; 200 mmol/l probe; 0.75 units Platinum Taq polymerase (Invitrogen); 200 mmol/l each of dATP, dCTP, dGTP and dTTP; 200 nmol/l ROX dye reference (Invitrogen); 16.6 mmol/l ammonium sulfate; 67 mmol/l Trizma (Sigma); 6.7 mmol/l magnesium chloride; 10 mmol/l mercaptoethanol; and 0.1% dimethyl sulfoxide. Duplicates of 3 ml of bisulfite-modified DNA solution were used in each real-time MSP amplification reaction. Primers and probes were designed to amplify a segment of a CpG island in the promoter of the genes of interest and of a reference gene, β-actin (ACTB), as previously described. Primers and probes were tested on positive (genomic methylated bisulfite converted DNA) and negative controls (genomic unmethylated bisulfite converted DNA) to ensure amplification of the desired product and non-amplification of unmethylated DNA, respectively. Primer and probe sequences and annealing temperatures are provided in Table II.

Amplification reactions were carried out in 384-well plates in a 7900 Sequence Detector (Perkin-Elmer Applied Biosystems) and were analyzed by SDS 2.2.1 (Sequence Detector System; Applied Biosystems). Thermal cycling was initiated with an initial denaturation step at 95°C for 3 min, followed by 50 cycles at 95°C for 15 sec and annealing temperature for 1 min. Each plate included patient DNA samples, positive (bisulfite converted hypermethylated universal DNA standard; Zymo Research), and multiple water blanks as non-template controls. Serial dilutions (60-0.006 ng) of this DNA were used to construct a calibration curve for each plate. The relative level of methylated DNA for each gene in each sample was determined as a ratio of qMSP for the amplified gene to ACTB and then multiplied by 100 for easier tabulation. The samples were categorized as unmethylated or methylated based on detection of methylation above a threshold set for each gene.

### Statistical analysis

Data were entered and compiled in the SPSS^®^ 22 statistical package (IBM SPSS, Chicago, IL, USA), following a standardized procedure to verify the data and correct for sample gaps or errors. Frequency distribution and cross-tabulation of selected variables were used to initially explore crude data, to evaluate the crude associations of each gene methylation value (along with other covariates) in regard to BC and DRC, and to explore the feasibility of further data analysis of each variable under study.

Methylation values were first analyzed as continuous variables and were also dichotomized into ‘methylated and not methylated’ using a cut-off identified by area under the curve (AUC) analyses (14,15). Unadjusted and adjusted analyses were used to examine the association between promoter methylation, BC and DRC levels, which were previously measured in this cohort with the host cell reactivation assay and using a luciferase reporter gene (11). In addition, the associations of all covariates with each gene methylation as compared to no methylation were an integral part of the analysis. After transforming continuous variables to approximate normality, we used the mean difference (differential methylation) to compare BC and control data. Mean differences in promoter methylation were also compared along high and low DRC levels. The 95% confidence intervals were used to assess the precision of the mean difference, and the Wilcoxon test was used to assess the statistical significance of the mean differences since the distribution of some of these variables was unknown or skewed. For categorical variables, the odds ratio (OR) was used as a measure of association, with a 95% confidence interval as an assessment of the precision of this estimate. The two-tailed Fisher’s exact test was calculated to measure the statistical significance of the crude OR.

We also explored confounding and interaction effects to establish associations between promoter methylation and BC, DRC level (low <4.97%; high ≥4.97%), and other covariates by means of the Mantel and Hansel stratified analysis. We used multiple logistic regressions to measure the adjusted OR and analyze possible interactions. After fitting the best model to adjust for potential confounders, we adjusted all associations by age, BMI, family history of BC, menopausal status, alcohol consumption, smoking status, multivitamin use and DRC.

## Results

### Cases and controls differ in several categories

Fifty-six percent of the cases and 38% of the controls were over 54 years of age; 73% of the cases and 63% of the controls had a BMI ≥25; 23% of the cases and 32% of the controls had a family history of BC; 29% of the cases and 17% of the controls had a history of alcohol consumption; 46% of the cases and 33% of the controls had a history of multivitamin use; 71% of the cases and 18% of the controls had high levels of DRC.

### Discovery cohort

Differential promoter methylation of *MAL*, *KIF1A*, *FKBP4*, *VGF* and *OGDHL* was examined by qMSP in the discovery cohort. We observed a significant association between *MAL* promoter methylation and BC (p=0.01), after adjusting for age, family history of cancer and DRC. A receiver operating curve analysis revealed that *MAL* promoter methylation had 94.1% sensitivity and 86.7% specificity; and 0.93 AUC.

The *MAL* promoter in women with BC had 44 times more odds to be methylated when compared to the controls. After adjusting for age and family history of BC, the OR increased from 44.0 to 46.3. Furthermore, after adjusting for DRC, the association increased almost 2-fold from 46.3 to 83.0. Promoter methylation of *KIF1A* (OR=21) and *FKBP4* (OR=5.9) was also associated with BC after adjusting for age, family history of BC, and DRC, albeit not significantly (Table III). We decided to drop *VGF* from further analyses since only 1 out of 18 cases was methylated.

The trend in the association between BC and promoter methylation of *MAL*, *KIF1A* and *FKBP4*, was strengthened in every case after adjusting for DRC, which led us to examine the association between promoter methylation of these genes and DRC. Table IV lists the crude and adjusted results of the association between promoter methylation and DRC in the discovery cohort. Women with low DRC had 3.0 and 3.3 times more odds to have *MAL* promoter and FKBP4 methylation respectively, and less likely to have *KIF1A* methylation, when compared to women with high DRC.

### Validation cohort

Promoter methylation of *MAL*, *KIF1A* and *OGDHL* was then quantified by qMSP in the validation cohort. Three of the four genes had receiver characteristic operator curve values of ≥0.50: *MAL* (0.64), *KIF1A* (0.51) and *OGDHL* (0.53). The mean of log-transformed promoter methylation values for *MAL*, *KIF1A* and *OGDHL* were higher in BC cases compared to the controls (Table V). The mean difference in log-transformed methylation values was highest for *OGDHL*, followed by *KIF1A*. This difference was statistically significant for *KIF1A* (p=0.032), borderline for *OGDHL* (p=0.096), and not significant for *MAL* (p=0.18). *KIF1A* (p=0.012) and *OGDHL* (p=0.048) were significantly associated with BC, after adjusting for age, family history of cancer and DRC.

We also found a significant difference in log transformed *KIF1A* promoter methylation (p=0.007) when comparing patients with high and low DRC (Table VI). Multivariate analysis showed that *KIF1A* promoter methylation was inversely associated with DRC after adjusting for age, BMI, family history of cancer, menopausal status, alcohol use, smoking, multivitamin use and BC (p=0.012).

## Discussion

We demonstrated that *KIF1A* promoter methylation can distinguish breast cancer (BC) cases from controls in plasma and was inversely associated with DNA repair capacity (DRC) levels in a BC case control study. *KIF1A* is directly involved in the microtubule-based transport of dense-core vesicles in mammalian neurons (16). Methylation of *KIF1A* is known to be frequent and show higher levels in thyroid cancer for example, when compared to normal thyroid tissue (17). Previous studies also showed it to be differentially hypermethylated (but not significantly associated with survival) in head and neck squamous cell carcinoma (HNSCC) (18). This was also found in plasma and saliva of lung cancer and HNSCC patients compared to controls, which suggests it could serve as a biomarker for early detection, particularly when used as part of a methylation panel of several genes (19,20). In BC, overexpression of *KIF1A* was found to correlate with chemotherapy resistance in cell lines (21).

The genes we analyzed in the present study, *MAL*, *FKBP4*, *KIF1A*, *VGF* and *OGDHL,* are differentially methylated in BC cell lines and tissue samples (7). *MAL* encodes a trans-membrane protein involved in the sorting of proteins for signaling and transport, and therefore is expressed in most types of epithelial cells (22). Epigenetic regulation of *MAL* in BC has been described and associated with silencing of expression. It is observed in up to 95% of BCs, more commonly in estrogen receptor (ER)- and progesterone receptor (PR)-positive tumors (7). *MAL* is methylated in other types of cancer as well (23,24). *FKBP4* is a component of a subclass of steroid receptor complexes, which by binding to heat shock protein 90 regulates the maturation of the receptor (25). Its expression was found to be upregulated in prostate cancer (26) and in ER-positive BCs (27). The *VGF* gene encodes a neuropeptide precursor with a restricted pattern of expression that is limited to a subset of neurons in the central and peripheral nervous systems and to specific populations of endocrine cells in the adenohypophysis, adrenal medulla, gastrointestinal tract and pancreas (28). *VGF* peptides play a multiplicity of roles including endocrine functions, local intercellular communication, as well as the possible mediation of intracellular mechanisms (29). *VGF* is also methylated in ovarian, testicular and head and neck tumors (8,30,31). *OGDHL* is part of the *OGDH* complex, whose malfunction is associated with neurodegeneration. Promoter methylation of the gene was noted in different tissue types. Cancer-specific methylation was evident in several types of cancer, including breast, while absent in others (32). The correlation between expression of the gene and proliferation properties of cells was also functionally demonstrated in cervical cancer cell lines.

Epigenetic changes, and specifically promoter methylation, have been well described in various types of tumors, and are known to be early events in cancer progression. Tumor-suppressor genes in tumors are frequently inactivated epigenetically by methylation when compared to normal tissue (33). However, the relationship between DRC and CpG promoter methylation has not previously been described in BC. Promoter methylation is involved in the pathogenesis of BC, and there is a constant attempt to utilize this for risk assessment, early detection, therapy monitoring among other applications, with no current consensus regarding the correct epigenetic alteration or combination of these. Recently, as part of the Cancer Genome Atlas Network project, methylation arrays were used to classify 802 BCs as part of a multiplatform BC subtype classification study (31). A cluster of tumors displayed a ‘hypermethylation phenotype’, in which 490 genes demonstrated promoter methylation associated with lower expression. This study, as well as ours, demonstrates an association between epigenetic and genetic mechanisms that contributes to cancer progression.

The concept of ‘liquid biopsies’ is under research and development in recent years, for early detection, treatment response and follow-up monitoring, in order to minimize patient burden and increase compliance while maintaining accuracy compared to tumor tissue biopsies (34). Several platforms have been suggested: blood, urine, saliva, nipple aspirate fluid for different types of cancers, using different assays - circulating tumor cells, genetic and epigenetic alterations and more. BC studies have compared the efficacy between tumor biopsy and detection of markers in plasma, including gene methylation, and have shown much promise, although, again, not reaching a consensus (8,35–37).

Interestingly, DRC was inversely correlated with *KIF1A* promoter hypermethylation. In this BC case-control study patients exhibited a decreased repair phenotype, on average 60% lower than age-matched controls (9,11). Epigenetic inactivation of DNA repair genes in cancer has been reported for several DNA repair pathways (33) including the nucleotide excision repair (NER) pathway (14), the pathway primarily measured by the host cell reactivation assay we used. The critical importance of the NER in BC was recently demonstrated. Mean NER capacity in BC tissue samples is significantly lower than that of normal breast tissue samples, averaging only 44% of normal activity (p<0.001) (38). Interestingly, the NER is the major human pathway for repairing a variety of bulky, helix-distorting DNA lesions, such as those induced by crosslinking agents and base-damaging carcinogens (39). Considered a ‘generalist’ of DNA repair pathways, the NER works in multiple capacities, particularly when other repair pathways exhibit reduced functionality (40).

Promoter methylation status is a simple test to conduct in plasma. The assessment of DRC is a more complicated procedure. This makes the identification of promoter methylation as surrogate molecular markers of DRC an interesting endeavor with translational applications. For example, promoter methylation of *KIF1A* could be used as a ‘treatment effectiveness’ biomonitor of DNA repair inhibitors, currently tested as potential therapeutic targets for BC subtypes (41–43).

A limitation of the present study is inherent in the nature of the assay used for DRC evaluation. It measures primarily the global activity of nucleotide excision repair genes involved in DNA repair, and therefore cannot pinpoint to specific genes within this pathway or to genes involved in other repair pathways that may be also associated with an increase in BC risk such as the NER.

In summary, we propose *KIF1A* methylation status as a potential biomarker for BC in plasma and a surrogate of DRC-related BC risk. The study design we utilized does not allow us to determine whether *KIF1A* promoter methylation is a driver of BC tumorigenesis or a passenger mark. Nor does it allow us to determine whether it is a biomarker of BC risk. We plan to perform functional studies to elucidate this relationship as we move forward in our understanding of promoter methylation and BC risk.

## Figures and Tables

**Table I:** Socio-demographic characteristics and risk factors of the study participants.

Variable	Cases n (%)	Controls n (%)	P-value	Total
Age (years)				
≤53	89 (44)	85 (62)	0.001^a^	174 (51)
>53	114 (56)	53 (38)		167 (49)
Mean ± SD	56±11.8	51±12.3	<0.001^a^	54.3±12.3
Median (range)	56 (30–89)	50 (22–86)		53 (22–89)
BMI				
≤25	54 (27)	51 (37)	0.042^a^	105 (30)
>25	149 (73)	86 (63)		235 (69)
Family history of BC				
Yes	32 (23)	41 (32)	0.505	73 (21)
No	106 (77)	86 (68)		268 (79)
Menopausal status				
Yes	25 (18)	35 (18)	>0.999	60 (18)
No	112 (82)	161 (82)		273 (82)
Alcohol consumption				
Yes	38 (29)	34 (17)	0.015	72 (21)
No	98 (71)	169 (83)		267 (79)
Smoking status				
Yes	22 (16)	32 (16)	>0.999	54 (16)
No	114 (84)	171 (84)		285 (84)
Multivitamin use				
Yes	63 (46)	67 (33)	0.023^a^	130 (38)
No	74 (54)	135 (67)		209 (62)
DRC				
Low	39 (29)	167 (82)	<0.001^a^	206 (60)
High	99 (71)	36 (18)		135 (40)

a Statistically significant.

BMI, body mass index; BC, breast cancer; DRC, DNA repair capacity.

**Table II:** qMSP primers and probe information.

Gene	Genbank #	Forward 5′-3′	Reverse 5′-3′	Annealing temp. °C
*ACTB*	Y00474	TGGTGATGGAGGAGGTTTAGTAAG T	AACCAATAAAACCTACTCCTCCCTTAA	60
*MAL*	NM_002371	GTTTTTAGTTTTGGACGTTCGTAG	CCAACCCCGCCCCCCGC	60
*KIF1A*	NM_004321	GCG CGA TAA ATT AGT TGG CGA TT	CTCGACGACTACTCTACGCTA T	58
*OGDHL*	NM_012446	TCGTTAGTATCGTGGATAGC	TACAAATCAAAAAACTACGCG	55
*VGF*	NM_003378	GGATAGCGTTCGTAGGCG	AAAAACCGAATTCCCCACCCCG	60

		Probe 6 FAM 5′-3′ TAMRA	Amplicon size (bp) (nucleotide range)	

*ACTB*		ACCACCACCCAACACACAATAACAAACACA	133 (390–522)	
*MAL*		AACACCGCCCTAAACCTCTTCGAAAC	104 (−204–100)	
*KIF1A*		CCTCCCGAAACGCTAATTAACTACGCG	140 (870–1010)	
*OGDHL*		CGCCGTACCAATTACCTAAATCAC	161 (881–1042)	
*VGF*		GCGCCCAAAAACGACGTAAACCTAAATAC	84 (−502--418)	

Temp., temperature.

**Table III:** Crude and adjusted ORs and 95% CIs for promoter methylation of the *MAL*, *KIF1A*, *FKBP4* and *OGDHL* genes in breast cancer cases and controls.

Gene	BC cases	Controls	Crude OR (95% CI)	P-value	Adjusted OR^a^ (95% CI)	P-value	Adjusted OR^b^ (95% CI)	P-value
*MAL*								
Methylated	16	4	44 (4.3–448.6)	<0.001^c^	46.3 (3.9–550.3)	0.002^c^	83 (2.8–2422.7)	0.01^c^
Not methylated	1	11						
*KIF1A*								
Methylated	3	2	1.6 (0.2–10.8)	>0.999	1.8 (0.3–12.7)	0.564	21 (0.5–819.6)	0.104
Not methylated	16	17						
*FKBP4*								
Methylated	4	1	4.8 (0.5–47.7)	0.340	4.2 (0.4–43.0)	0.229	5.9 (0.3–130.1)	0.258
Not methylated	15	18						
*OGDHL*								
Methylated	4	0	7.0^d^ (1.56e-60–3.15e+61)	0.978	6.7^d^ (1.24e-60–3.62e+61)	0.979	6.5^d^ (8.61e-61–4.94e+61)	0.979
Not methylated	15	19						

a Adjusted by age and family history of breast cancer;

b adjusted by age, family history of breast cancer and DRC.

c Statistically significant results.

d Only cases are methylated, thus GLM was used to estimate odds, risk, by exponentiation of the appropriate coefficients.

BC, breast cancer; OR, odds ratio; CI, confidence interval.

**Table IV:** Promoter methylation of the *MAL*, *KIF1A*, *FKBP4* and *OGDHL* genes in breast cancer patients with low and high DNA repair capacity (DRC).

Gene	Low DRC <4.97%	High DRC ≥4.97%	Crude OR (95% CI)	P-value	Adjusted OR^a^ (95% CI)	P-value
*MAL*						
Methylated	14	6	3.2 (0.7–15.6)	0.150	3 (0.6–14.6)	0.182
Not methylated	5	7				
*KIF1A*						
Methylated	2	3	0.4 (0.06–2.96)	0.632	0.4 (0.06–3.0)	0.385
Not methylated	20	13				
*FKBP4*						
Methylated	4	1	3.3 (0.3–33.1)	0.374	3.3 (0.3–34.0)	0.319
Not methylated	18	15				
*OGDHL*						
Methylated	4	0	0.1^b^ (3.06e−62–7.28e+59)	0.979	0.2^b^ (1.77e−62–1.99e+60)	0.980
Not methylated	18	16				

a Adjusted by age and family history of breast cancer.

b Only cases are methylated, thus GLM was used to estimate odds, risk, by exponentiation of the appropriate coefficients.

OR, odds ratio; CI, confidence interval.

**Table V:** Mean methylation values, mean differences, and crude and adjusted ORs and 95% CIs for promoter methylation of the *MAL*, *KIF1A*, *FKBP4* and *OGDHL* genes in breast cancer cases and controls.

Gene	Cases mean (n)	Controls mean (n)	Mean difference (95% CI)	P-value	Crude OR (95% CI)	P-value	Adjusted OR^a^ (95% CI)	P-value	Adjusted OR^b^ (95% CI)	P-value
*MAL*	1.5 (154)	1.4 (86)	−0.1 (−0.2–0.04)	0.180	1.4 (0.8–2.4)	0.179	1.4 (0.8–2.5)	0.257	1.3 (0.7–2.6)	0.407
*KIF1A*	1.6 (50)	1.4 (38)	−0.2 (−0.4, −0.02)	0.032^c^	0.4 (0.2–0.9)	0.033^c^	3.8 (1.3–10.8)	0.012^c^	2.4 (0.7–8.3)	0.169
*OGHDL*	1.6 (30)	1.3 (15)	−0.3 (−0.6–0.1)	0.096	3.0 (0.8–11.0)	0.097	5.2 (1.1–27.1)	0.048^c^	-	0.992

a Adjusted by age, BMI, family history of breast cancer, menopausal status, alcohol use, smoking status and multivitamin use;

b adjusted by age, BMI, family history of breast cancer, menopausal status, alcohol use, smoking, multivitamin use and DRC.

c Indicates statistically significant results.

OR, odds ratio; CI, confidence interval; BMI, body mass index; DRC, DNA repair capacity.

**Table VI:** Mean methylation values, mean differences and crude and adjusted ORs and 95% CIs for promoter methylation of the *MAL*, *KIF1A*, *FKBP4* and *OGDHL* genes in breast cancer cases with low and high DRC levels.

Gene	Low DRCs mean (n)	High DRCs mean (n)	Mean difference (95% CI)	P-value	Crude OR 95% (CI)	P-value	Adjusted OR^a^ 95% (CI)	P-value	Adjusted OR^b^ 95% (CI)	P-value
*MAL*	−3.3 (154)	−3.6 (82)	0.3 (−0.2–0.7)	0.27	0.8 (0.5–1.4)	0.392	0.8 (0.4–1.4)	0.396	0.9 (0.4–1.7)	0.726
*KIF1A*	−3.9 (49)	−5.2 (33)	1.3 (0.4–2.3)	0.007^b^	0.3 (0.1–0.7)	0.009^b^	0.1 (0.04–0.5)	0.002^b^	0.1 (0.04–0.7)	0.015^b^
*OGHDL*	−5.3 (26)	−5.6 (17)	0.6 (−0.9–1.6)	0.603	0.4 (0.1–1.5)	0.194	0.3 (0.1–1.3)	0.112	0.7 (0.1–6.6)	0.728

a Adjusted by age, BMI, family history of breast cancer, menopausal status, alcohol use, smoking status and multivitamin use;

b adjusted by age, BMI, family history of breast cancer, menopausal status, alcohol use, smoking status, multivitamin use and BC.

c Indicates statistically significant results.

OR, odds ratio; CI, confidence interval; BMI, body mass index.
